# Supplementary material for: The γ-tubulin meshwork assists in the recruitment of PCNA to chromatin in mammalian cells
Source: Commun Biol. 2021 Jun 22;4:767. doi: 10.1038/s42003-021-02280-1 (PMC8219688; doi:10.1038/s42003-021-02280-1)
Supplement: Supplementary file 2 — Supplemental Information [file 42003_2021_2280_MOESM2_ESM.pdf]

Number of precipitated origins of replication		
ChIP-seq	active	dormant
nonsynchronous	60	38
3h	72	60
7h	1	28
9h	84	25

Peaks overlapping with origins of replication				
ChIP-seq	Nr active domain	% active	Nr dormant domain	% dormant
nonsynchronous				
TUBG	55	91.7	29	76.3
FoxM1	51	85.0	16	42.1
3h				
TUBG	71	98.6	19	31.7
FoxM1	54	82.7	19	31.7
7h				
TUBG	1	100.0	14	50.0
FoxM1	1	100.0	6	21.4
9h				
TUBG	83	98.8	15	60.0
FoxM1	56	66.7	6	24.0

Supplementary Table 1. **List of the number of replication origins and of the γ-tubulin and FoxM1 peaks that overlap with either active or dormant origin of replication**. Mcm5 peaks that overlap with PCNA peaks (by at least 25% for both peaks) were considered active origins and those not overlapping with PCNA as dormant. The number and percentage of active (Nr active domain; % active) and dormant (Nr peaks in dormant; % dormant) domains with at least one peak of γ-tubulin or FoxM1 is indicated.

Tumor types	*TUBG1/PCNA*	*TUBG2/PCNA*
Adrenocortical adenocarcinoma	pvalue < 0.001 - R: 0.45	pvalue 0.15 - R: 0.17
Bladder urothelial carcinoma	pvalue < 0.001 - R: 0.63	pvalue 0.021 - R: -0.11
Breast invasive carcinoma	pvalue < 0.001 - R: 0.38	pvalue < 0.001 - R: -0.11
Cervical squamous cell carcinoma	pvalue < 0.001 - R: 0.33	pvalue 0.49 - R: 0.04
Cholangiocarcinoma	pvalue < 0.001 - R: 0.54	pvalue 0.11 - R: 0.27
Colon adenocarcinoma	pvalue < 0.001 - R: 0.24	pvalue < 0.001 - R: -0.21
Diffuse large B cell lymphoma	pvalue < 0.001 - R: 0.77	pvalue 0.11 - R: 0.24
Esophagal carcinoma	pvalue < 0.001 - R: 0.37	pvalue 0.24 - R: 0.0087
Glioblastoma multiforme	pvalue < 0.001 - R: 0.6	pvalue 0.2 - R: 0.1
Head and neck squamous carcinoma	pvalue < 0.001 - R: 0.39	pvalue 0.013 - R: 0.11
Chromophobe renal cell carcinoma	pvalue < 0.001 - R: 0.72	pvalue 0.3 - R: 0.13
Clear cell renal cell carcinoma	pvalue < 0.001 - R: 0.47	pvalue 0.11 - R: 0.071
Papillary renal cell carcinoma	pvalue < 0.001 - R: 0.44	pvalue < 0.001 - R: 0.24
Acute myeloid leukemia	pvalue < 0.001 - R: 0.58	pvalue 0.094 - R: 0.13
Low grade glioma	pvalue < 0.001 - R: 0.71	pvalue < 0.001 - R: -0.24
Hepatocellular carcinoma	pvalue < 0.001 - R: 0.64	pvalue < 0.001 - R: 0.38
Lung adenocarcinoma	pvalue < 0.001 - R: 0.51	pvalue 0.031 - R: 0.098
Lung squamous cell carcinoma	pvalue < 0.001 - R: 0.38	pvalue 0.1 - R: 0.075
Mesothelioma	pvalue < 0.001 - R: 0.69	pvalue 0.66 - R: 0.047
Ovarian serous cystadenocarcinoma	pvalue < 0.001 - R: 0.45	pvalue 0.0019 - R: 0.15
Pancreatic adenocarcinoma	pvalue < 0.001 - R: 0.55	pvalue 0.24 - R: -0.088
Pheochromocytoma	pvalue < 0.001 - R: 0.55	pvalue < 0.001 - R: 0.36
Prostate adenocarcinoma	pvalue < 0.001 - R: 0.43	pvalue 0.34 - R: -0.044
Rectum adenocarcinoma	pvalue < 0.001 - R: 0.46	pvalue 0.39 - R: -0.091
Sarcoma	pvalue < 0.001 - R: 0.62	pvalue 0.0023 - R: 0.19
Skin cutaneous melanoma	pvalue < 0.001 - R: 0.35	pvalue 0.0023 - R: 0.16
Stomach adenocarcinoma	pvalue < 0.001 - R: 0.38	pvalue < 0.001 - R: -0.19
Testicular germ cell tumors	pvalue < 0.001 - R: 0.5	pvalue 0.15 - R: -0.12
Thyroid carcinoma	pvalue < 0.001 - R: 0.39	pvalue 0.71 - R: 0.016
Thymoma	pvalue < 0.001 - R: 0.44	pvalue < 0.001 - R: -0.37
Endometrial carcinoma	pvalue < 0.001 - R: 0.65	pvalue < 0.001 - R:-0.47
Uterine carcinosarcoma	pvalue < 0.001 - R: 0.32	pvalue 0.66 - R: 0.06
Uveal melanoma	pvalue < 0.001 - R: 0.49	pvalue 0.014 - R: 0.42

Supplementary Table 2. List of the 33 subtypes of tumors found in the employed TCGA datasets containing information on 9664 tumors, Related to Figures 7 and S8. RNA expression of *TUBG1* in tumor material displays a positive correlation to *PCNA* expression, but not to the expression of *TUBG2*. R represents the Pearson product moment correlation coefficient between the indicated RNA expression with respective p-values.

A. Primer used for mutagenesis, mutated or inserted bases underlined			
Gene	Mutation	Forward primer	Reverse primer
TUBG2	Deletion of *Eco*RI	GCATCGTGGAGGAGTTCGCCAC CGAGG	CCTCGGTGGCGAACTCCTCCAC GATGC
TUBG1	Q429A–I432A–Y435A	GGAGATTGTGGCGCAGCTCGCC GATGAGGCCCATGCGGC	GCCGCATGGGCCTCATCGGCGA GCTGCGCCACAATCTCC

B. Primer used for cloning single guide RNA		
Gene	Forward primer	Reverse primer
PCNA	CACCGGGACTCGTCCCACGTCTCTT	AAACAAGAGACGTGGGACGAGTCCC

C. Primer used for amplification		
Gene targeted	Forward primer	Reverse primer
TUBG2	GCGGAATTCACCATGCCCCGGGAGATCATC ACCCTG	GCGGATATCTCACTGCTCCTGGGTG CCCCAGGAA

D. Primer used for detection of PURA DNA-binding motif in chromosome 4		
Amplified region	Forward primer	Reverse primer
49109512-491097020	GTTCTGTTCCACTCCATTCCATTG	GAAAGGAACGGAATGGAATCAACC

Supplementary Table 3. Description of primers used in this study

A. Modified cell lines				
Cell line	Expressing	Co-expressing	Selection	Denoted
U2OS	*TUBG1* sgRNA Cas9-crispGFP	–	GFP	*TUBG1*-sgRNA-U2OS
U2OS	*TUBG1* sgRNA Cas9-crispGFP	*TUBG2*	GFP, G418	*TUBG1*-sgRNA-U2OS-TUBG2
U2OS	*TUBG1* sgRNA Cas9-crispGFP	*TUBG1*	GFP, G418	*TUBG1*-sgRNA-U2OS-TUBG1
U2OS	*PCNA* sgRNA Cas9-crispGFP	–	GFP	*PCNA*-sgRNA-U2OS
U2OS	*TUBG* shRNA	–	Zeocin,	*TUBG*-shRNA-U2OS
MCF10A	*TUBG* shRNA	–	Zeocin	*TUBG*-sh-MCF10A
U2OS	*TUBG* shRNA	GFP-γ-tubulin	Zeocin G418	*TUBG*-shRNA-U2OS-TUBG1-GFP
U2OS	*TUBG* shRNA	GFP-γ-tubulin$_{resist}$, CCC	Zeocin, G418	*TUBG*-shRNA-U2OS-TUBG1-CCC
U2OS	*TUBG* shRNA	GFP-N-γ-tubulin$^{1-335}$	Zeocin, G418	*TUBG*-shRNA-U2OS-TUBG1$^{1-335}$-GFP
U2OS	*TUBG* shRNA	GFP-C-γ-tubulin$^{336-451}$	Zeocin, G418	*TUBG*-shRNA-U2OS-TUBG1$^{336-451}$-GFP
U2OS	*TUBG* sgRNA Cas9-crispGFP	*TUBG1*$^{A429-A432-A435}$	GFP, G418	*TUBG1*-sgRNA-U2OS-TUBG1$^{A429-A432-A435}$
U2OS	*TUBG1* sgRNA Cas9-crispGFP	*TUBG1*, CCC	GFP, G418	*TUBG1*-sgRNA-U2OS-TUBG1-CCC
U2OS	*TUBG1* sgRNA Cas9-crispGFP	*TUBG1*$^{A429-A432-A435}$, CCC	GFP, G418	*TUBG1*-sgRNA-U2OS-TUBG1$^{A429-A432-A435}$-CCC

Supplementary Table 4. Description of modified cell lines used in this study

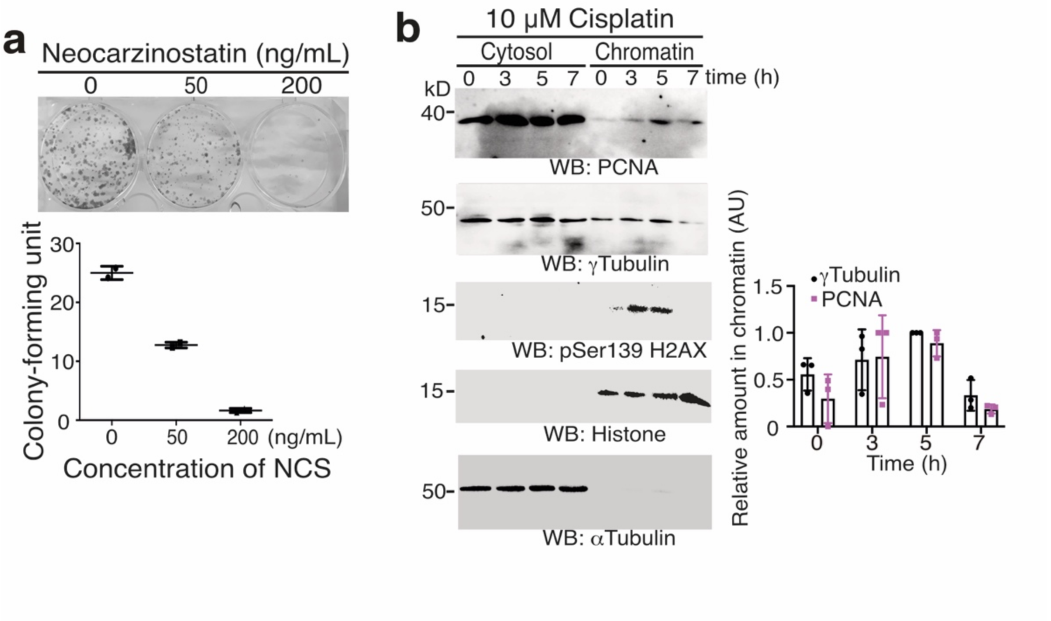

Supplementary Figure 1. Dose response analysis of NCS and cellular response to cisplatin. **a** The image and graphic show the colony forming unit (percentage of colonies formed) after 2 weeks of culture of U2OS cells treated with various concentrations of neocarzinostatin ($N = 3$). **b** Cisplatin-treated U2OS cells (1.0 x 10^6) were lysed, and proteins were biochemically divided into cytosolic (Cytosol), and chromatin fractions and then analyzed by western blotting (blots designated WB) with an anti-PCNA antibody, and anti-γ-tubulin, anti- phospho-histone2AX (marker of DSB), anti-α-tubulin, and anti-histone antibodies ($N = 3$). **a, b** Source data are provided as a Source Data file.

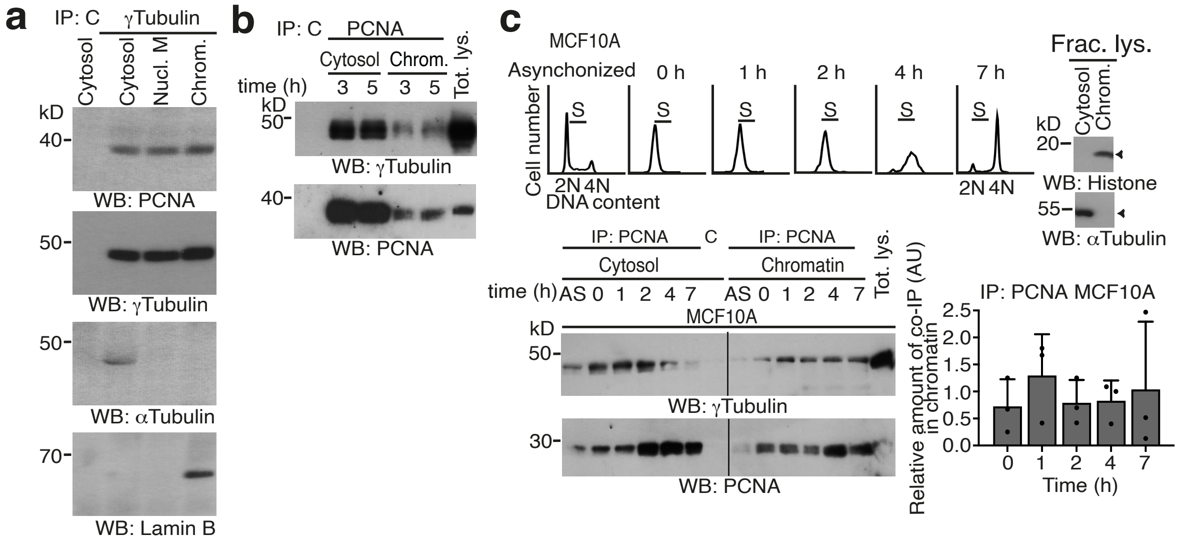

Supplementary Figure 2. Analysis of γ-tubulin and PCNA immunoprecipitates in mammalian cell lines. **a** γ-Tubulin co-precipitates with PCNA in all studied biochemical fractions. In extracts from non-synchronous NIH3T3 cells biochemically divided into cytosolic (Cyt), nuclear membrane (Nucl. M), and chromatin (Chrom.) fractions, the γ-tubulin was immunoprecipitated with an anti-γ-tubulin antibody, developed by WB with an anti-PCNA antibody (top) and reprobed with α-tubulin and laminB, as indicated ($n = 3$). LaminB and α-tubulin were used as controls for the different compartments. An isotype-matched control antibody (C) was used in cytosolic lysates. **b, c** Using extracts from S phase synchronized U2OS (**b**) or MCF10A (**c**) cells, γ-tubulin or PCNA was immunoprecipitated with an anti-γ-tubulin,

an anti-PCNA or an isotype-matched control (C; 5 h or 2 h cytosolic lysates) antibody, and developed by WB with the indicated antibodies (N = 3). The top panels illustrate the DNA content of the cells. Fractionated lysates (Frac. lys.) were run as control for the biochemical fractionation (right) and analyzed by WB using α-tubulin and histone as molecular markers for the cytosolic and nuclear fractions, respectively. **c** The graph illustrates densitometric analysis of the γ-tubulin chromatin content in the analyzed WBs. To adjust for differences in protein loading, the concentration of a protein was determined as its ratio with the immunoprecipitated protein for each time point. **a–c** Source data are provided as a Source Data file.

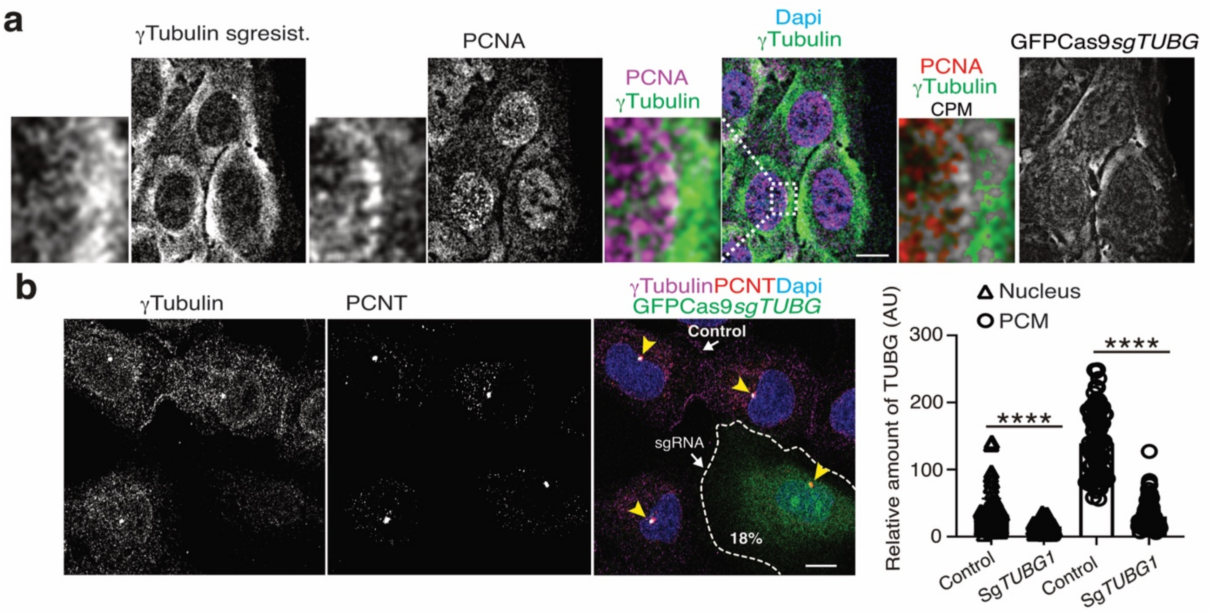

Supplementary Figure 3. Determining the location of PCNA and pericentrin. **a**, **b** Localization of PCNA (**a**), pericentrin (PCNT; **b**) and γ-tubulin (**a**, **b**) was examined by immunofluorescence staining with an anti-PCNA, anti-PCNT and anti-γ-tubulin (**a**: rabbit T3320; **b**: mouse T6557) antibodies in U2OS cells co-expressing human *TUBG*-sgRNA (GFP-Cas9*sgTUBG*) and sg-resistant *TUBG1* (γTubulin sgresist.) or expressing human GFP-Cas9*sgTUBG*, as indicated. Nuclei were detected with DAPI. The white box and arrowheads indicate the magnified area shown in the insets and centrosomes, respectively (N = 3). Colocalization pixel-map (CPM) represents the magenta and green channels of the magnified areas displayed in the inset. White areas denote colocalized pixels between channels. Scale bar: 10 μm. **b** Within samples, quantification of γ-tubulin signal in a nucleus or periocentriolar matrix (PCM) was done with ImageJ software by comparison of immunofluorescence-labelled γ-tubulin in cells expressing Cas9-crispGFPsg*TUBG1* (dashed lines, N = 100 cells) with non-expressing cells (control, N = 100 cells). The graph illustrates the mean value of the γ-tubulin content in the nucleus or PCM (mean ± SD; ****P < 0.0001). Numbers in images indicate the remaining protein levels of γ-tubulin relative to control in the indicated cell.

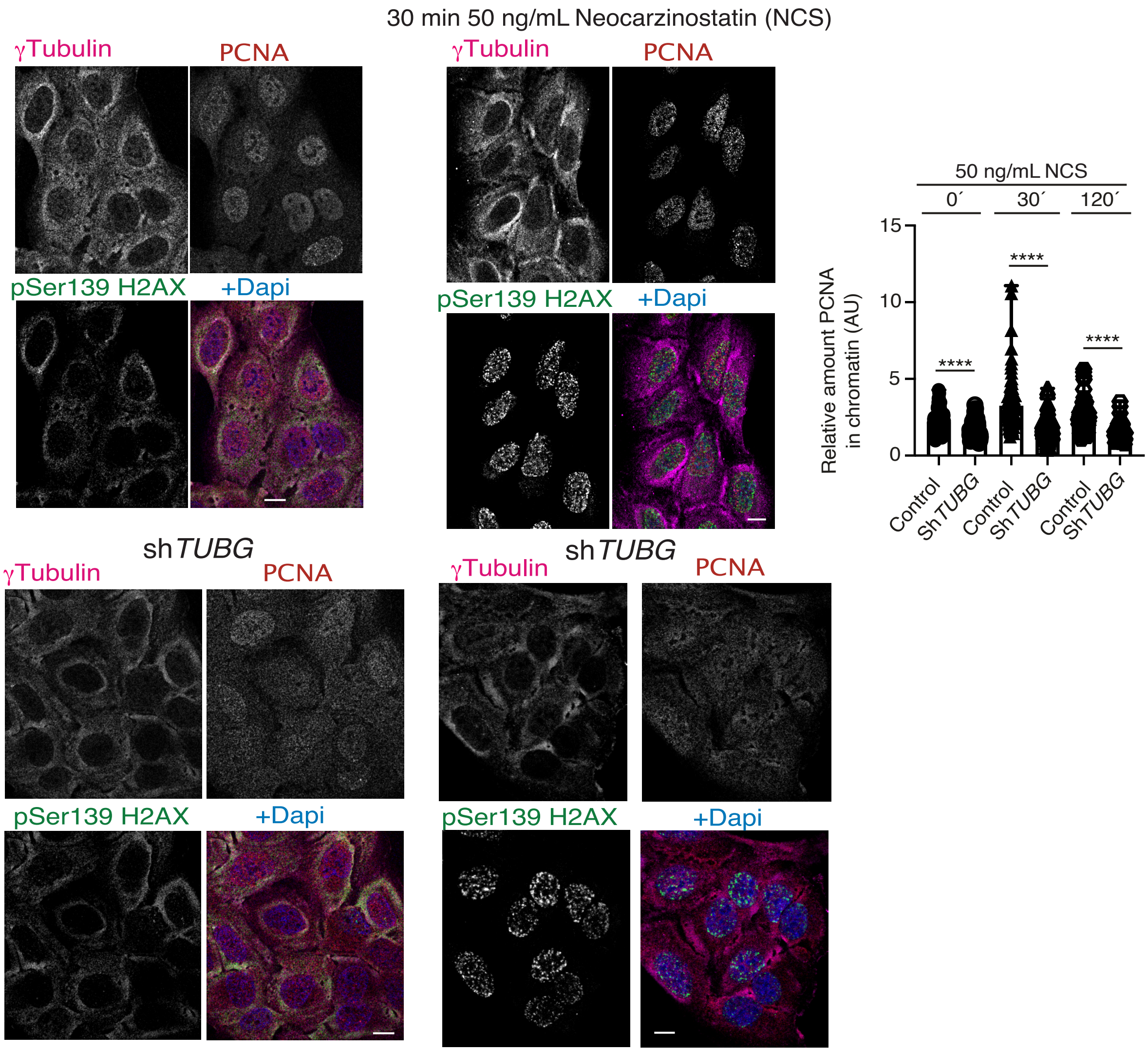

Supplementary Figure 4. Reduced levels of γ-tubulin decrease the chromatin loading of PCNA after treatment with neocarzinostatin. Localization of endogenous PCNA was examined by immunofluorescence staining with an anti-PCNA and the indicated antibodies, and nuclei were detected with DAPI, in control U2OS cells or U2OS cells stably expressing *TUBG* short hairpin (sh)RNA after treated with neocarzinostatin (NCS) for 2 h. Scale bars: 10 μm ($N = 3$). Within samples, quantification of the PCNA signal in chromatin was done with ImageJ software by comparison of immunofluorescence-labelled PCNA in non-treated U2OS (control, $N = 104$ cells) with U2OS cells stably expressing sh*TUBG* ($N = 112$ cells) and in NCS-treated control (control, $N = 98$ cells) with NCS-treated sh*TUBG* cells ($N = 99$ cells) . The Graph illustrates the mean value of PCNA in chromatin (mean ± SD; ****$P < 0.0001$).

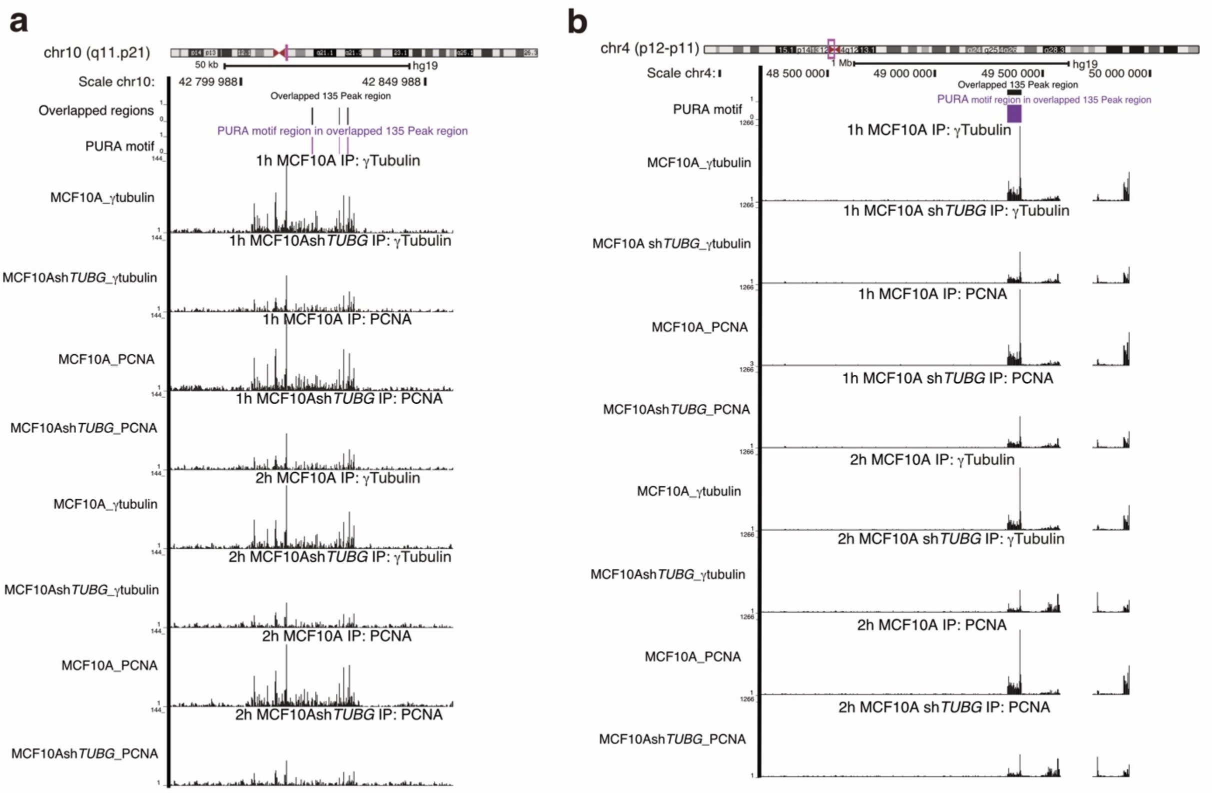

Supplementary Figure 5. PCNA and γ-tubulin are frequently localized to the same DNA region. a,b MCF10A and MCF10A cells stably expressing *TUBG* shRNA (MCF10Ash*TUBG*) were synchronized in early S phase (0 h) and released for 1 and 2 h. To map the DNA region where γ-tubulin accumulates in the chromatin, we sequenced the DNA associated with chromatin immunoprecipitates from γ-tubulin and PCNA. The diagram illustrates ChIP-seq analysis of γ-tubulin and PCNA distribution on chromosome 10 (Chr10, **a**) and chromosome 4 (Chr4, **b**) in the indicated area (magenta line and box; $N = 2$).

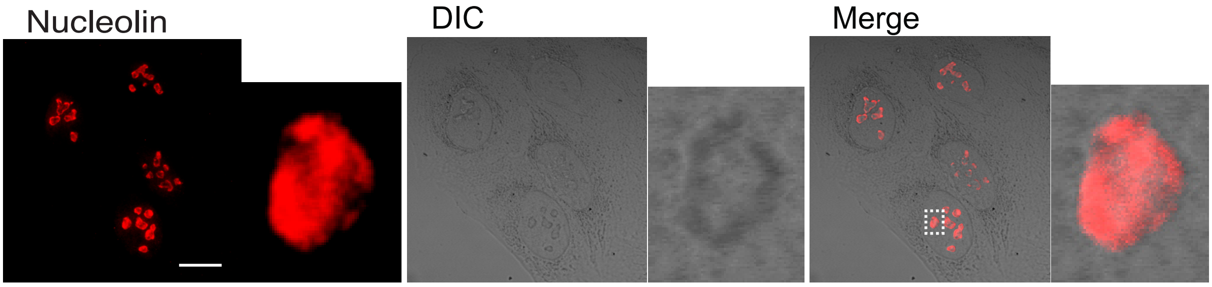

Supplementary Figure 6. Determining the location of nucleoli. Localization of nucleoli was examined by immunofluorescence staining with an anti-nucleolin antibody in U2OS cells. Scale bar: 10 μm.

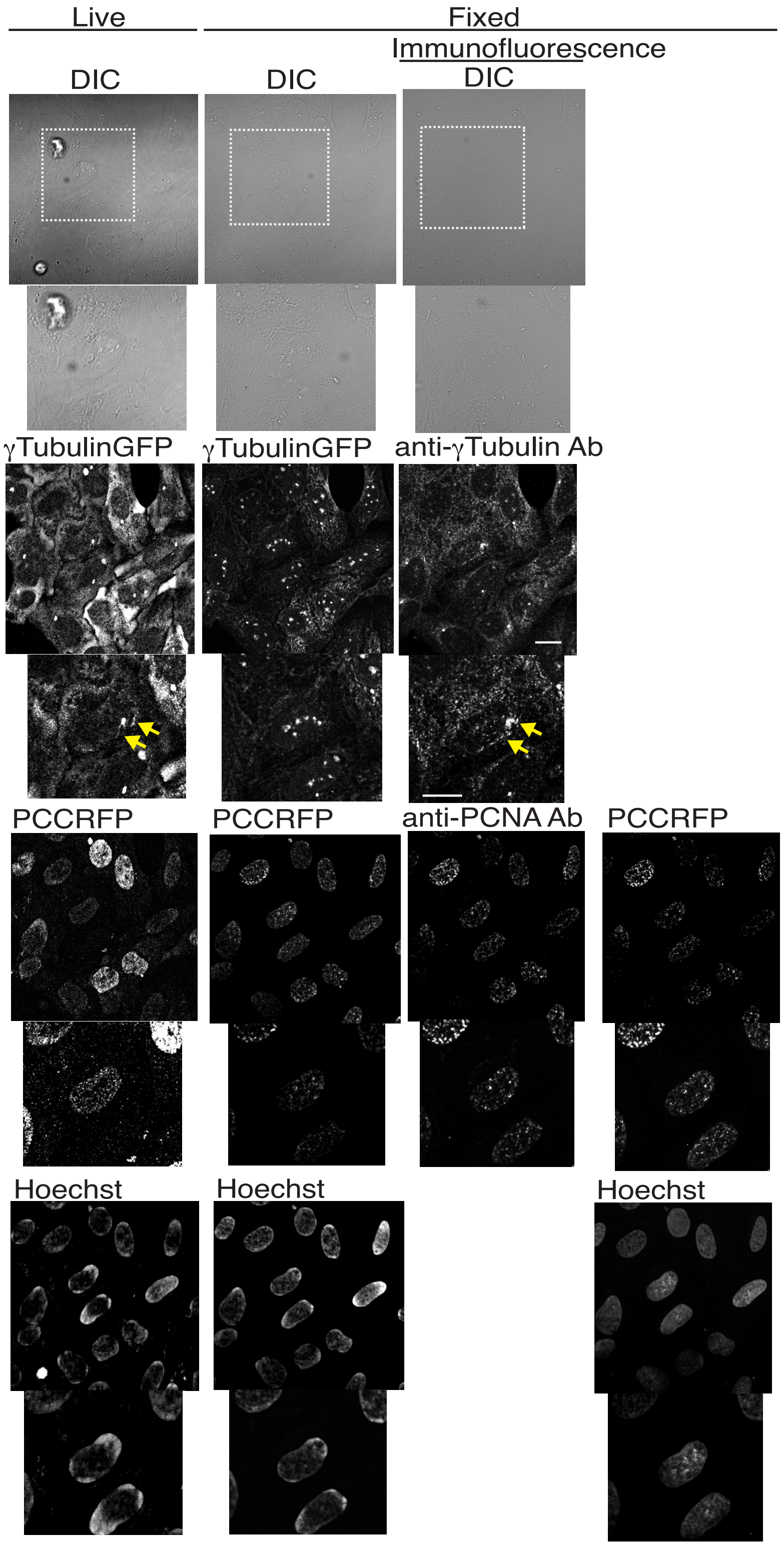

Supplementary Figure 7. Comparison of the γ-tubulin meshwork in fixed and immunostained cells and live cells. The DIC/fluorescence images present the U2OS cell shown in Fig. 5c. After time-lapse imaging, the U2OS cell was fixed and immunofluorescence stained with an anti-γ-tubulin and an anti-PCNA antibody, and the nuclei were stained with Hoechst. Yellow arrows indicate γ-tubules. Scale bars: 10 μm.

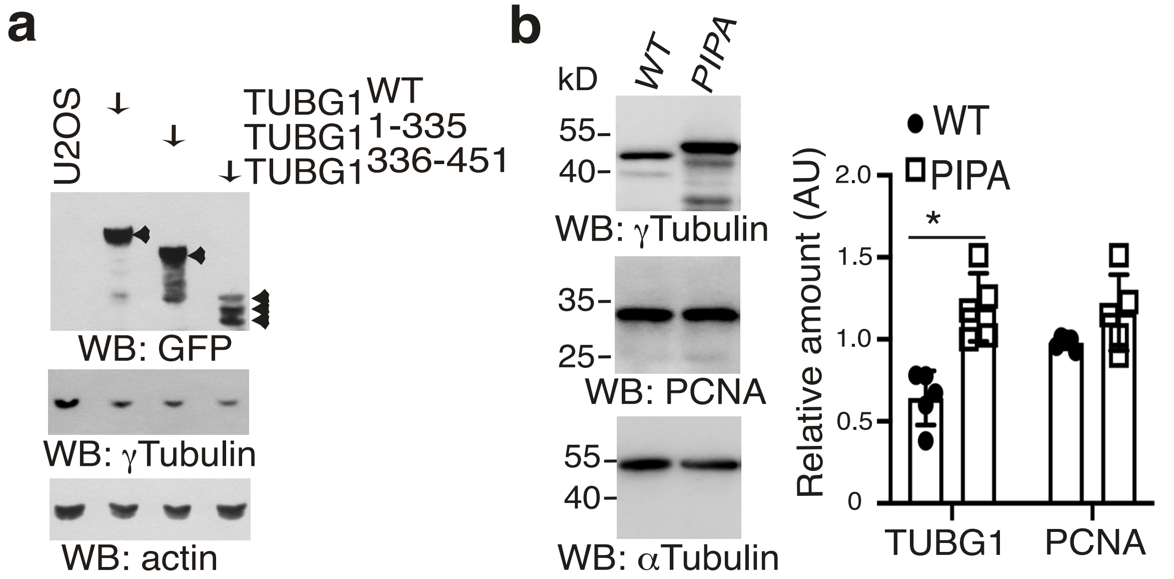

Supplementary Figure 8. Levels of ectopically expressed proteins. **a** Cytosolic extracts from U2OS cells, or U2OS cells stably coexpressing *TUBG*-shRNA and either GFP-γ-tubulin, GFP-N-γ-tubulin$^{1\text{-}335}$, or GFP-C-γ-tubulin$^{336\text{-}451}$ were used, and analyzed by WB for the expression of recombinant GFP-tagged proteins and endogenous γ-tubulin. An actin loaded control is shown ($N = 3$). Arrowheads indicate the various GFP-fused proteins. **b** The cytosolic fraction from a stable *TUBG*sg-U2OS cell co-expressing γ-tubulin$_{resist}$ (WT) or a γ-tubulin$^{A429\text{-}A432\text{-}A435}_{resist}$ (PIPA) was analyzed by WB for the expression of recombinant γ-tubulin and endogenous PCNA. An α-tubulin loading control is also shown. The graph illustrates the mean value of γ-tubulin and of the PCNA signal in chromatin (mean ± sd.; $N = 5$, $^*P < 0.05$). To adjust for differences in protein loading, the concentration of a protein was determined as its ratio with α-tubulin for each time point. **a, b** Source data are provided as a Source Data file.

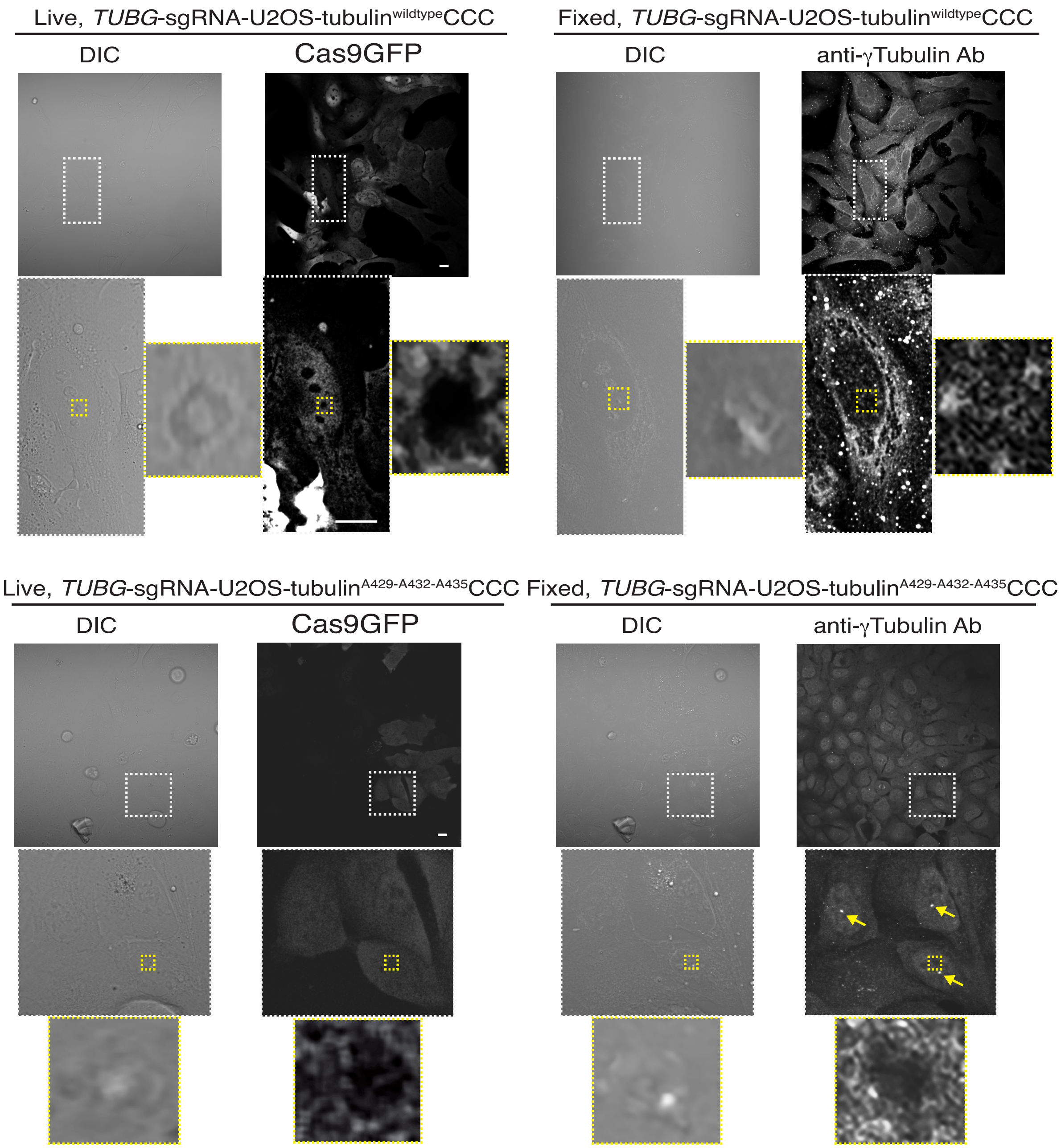

Supplementary Figure 9. Determining the location of γ-tubulin in fixed and immunostained cells after live imaging. The DIC/fluorescence images illustrate the stable *TUBG*sg-U2OS cells shown in Fig. 7, co-expressing either γ-tubulin$_{resist}$ or a γ-tubulin$^{A429\text{-}A432\text{-}A435}_{resist}$, as indicated. After time-lapse imaging, the U2OS cells were fixed and immunofluorescence stained with an anti-γ-tubulin. Yellow arrows indicate centrosomes. Scale bars: 10 μm.

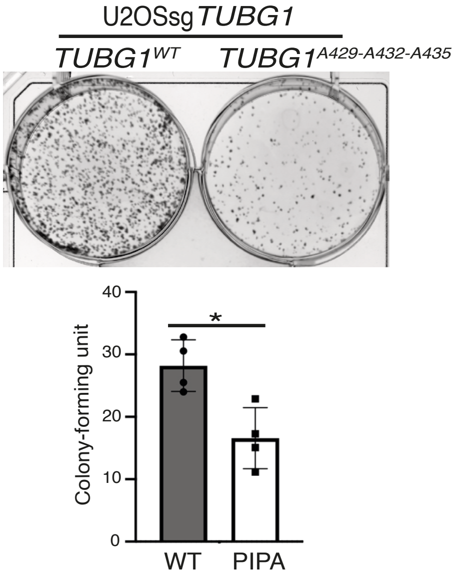

Supplementary Figure 10. The colony-forming unit capacity of cells expressing TUBG1$^{A429-A432-A435}$. The image and the graph show the colony-forming unit (percentage of colonies formed) of a stable *TUBG*sg-U2OS (U2OSsg*TUBG1*) cell line co-expressing single-guided (sg)-resistant γ-tubulin (TUBG1^{WT}; WT) or a γ-tubulin$^{A429-A432-A435}$ (TUBG1$^{A429-A432-A435}$; PIPA) after 2 weeks of culture (mean ± SD.; $N = 4$, *$P < 0.05$). Source data are provided as a Source Data file.

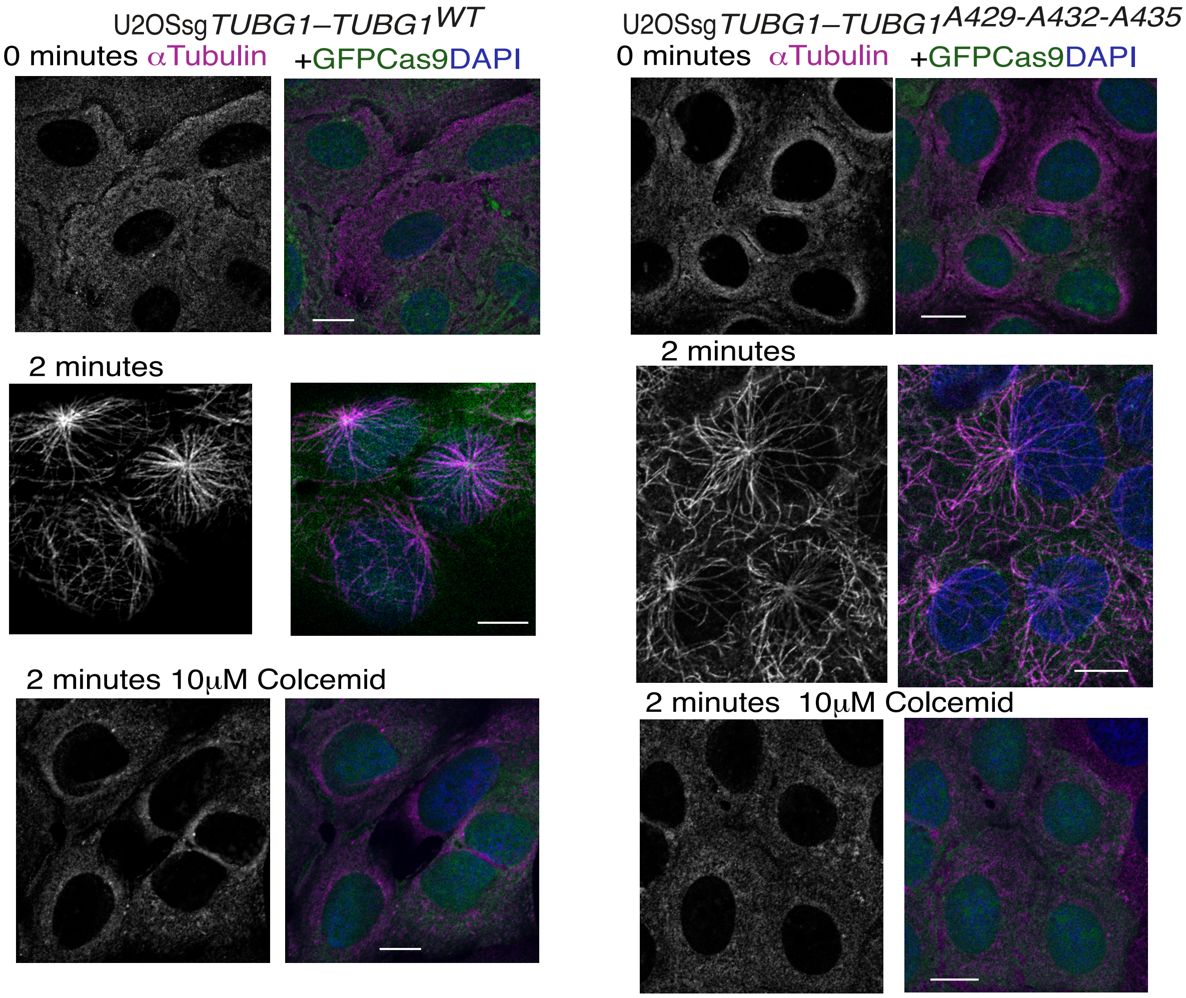

Supplementary Figure 11. γ-Tubulin$^{A429\text{-}A432\text{-}A435}$ does not alter microtubule nucleation. α-Tubulin in U2OS cells stably co-expressing *TUBG*-sgRNA and either γ-tubulin$_{resist}$ or a γ-tubulin$^{A429\text{-}A432\text{-}A435}{}_{resist}$ were stained immediately following cold treatment (0 min), 2 min after adding warm medium, or in the presence of 100 ng/mL colcemid (0°C for 30 min followed by 37°C for 2 min; impairs microtubule regrowth), as indicated. Scale bars: 10 µm.

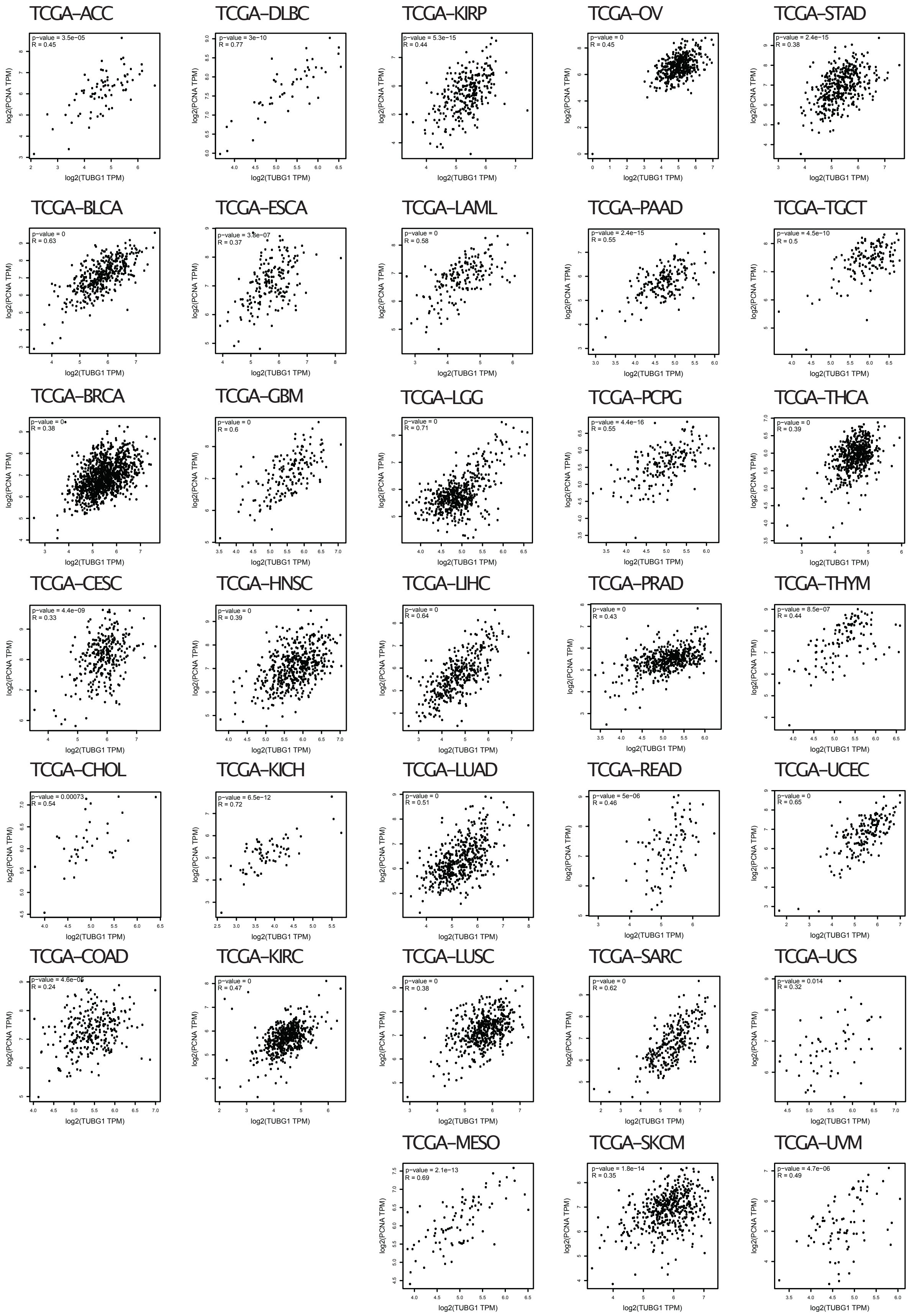

Supplementary Figure 12. RNA expression of *TUBG1* in tumor material shows a positive correlation with *PCNA* expression. Scatter plot of log2 *TUBG1* expression vs. log 2 *PCNA* expression in 33 different types of tumors. In the displayed plots, R represents the Pearson

product moment correlation coefficient between *TUBG1* and *PCNA* expression with respective p-values. For more information, please see Supplemental Table 1. ACC (adrenocortical carcinoma), BLCA (bladder urothelial carcinoma, BRCA (breast invasive carcinoma), CESC (cervical squamous cell carcinoma and endocervical adenocarcinoma), CHOL (cholangiocarcinoma), COAD (colon adenocarcinoma), DLBC (lymphoid neoplasm diffuse large B-cell lymphoma), ESCA (esophageal carcinoma), GBM (glioblastoma multiforme), HNSC (head and neck squamous cell carcinoma), KICH (kidney chromophobe), KIRC (kidney renal clear cell carcinoma), KIRP (kidney renal papillary cell carcinoma), LAML (acute myeloid leukemia), LGG (brain lower grade glioma), LIHC (liver hepatocellular carcinoma), LUAD (lung adenocarcinoma), LUSC (lung squamous cell carcinoma), MESO (mesothelioma), OV (ovarian serous cystadenocarcinoma), PAAD (pancreatic adenocarcinoma), PCPG (pheochromocytoma and paraganglioma), PRAD (prostate adenocarcinoma), READ (rectum adenocarcinoma), SARC (sarcoma), SKCM (skin cutaneous melanoma), STAD (stomach adenocarcinoma), TGCT (testicular germ cell tumors), THCA (thyroid carcinoma), THYM (thymoma), UCEC (uterine corpus endometrial carcinoma), UCS (uterine carcinosarcoma), UVM (uveal melanoma).
