# Supplementary material for: The γ-tubulin meshwork assists in the recruitment of PCNA to chromatin in mammalian cells
Source: Commun Biol. 2021 Jun 22;4:767. doi: 10.1038/s42003-021-02280-1 (PMC8219688; doi:10.1038/s42003-021-02280-1)
Supplement: Supplementary file 3 — Description of Additional Supplementary Files [file 42003_2021_2280_MOESM3_ESM.pdf]

Description of Additional Supplementary Files

File Name: Supplementary Data 1
Description: Includes raw data to the manuscript

File Name: Supplementary Movie 1
Description: Fluorescence images from a time-lapse Z-stack series using a laserscanning confocal microscopy of a stable TUBGsh-U2OS cell co-expressing GFP-γtubulinresist (γTubulinGFP) and cell cycle chromobody (CCCRFP, to visualize the nuclear location of endogenous PCNA in a living cell). The movie show the resulting average intensity projection of ten sequential images that were collected at 0.4-µm intervals. Z-stack series were collected every 5 minutes.

File Name: Supplementary Movie 2
Description: Fluorescence images from a time-lapse confocal microscopy using a laser-scanning confocal microscopy of a stable TUBGsh-U2OS cell co-expressing GFP-γtubulinresist (γTubulinGFP) and cell cycle chromobody (CCCRFP, to visualize the nuclear location of endogenous PCNA in a living cell). Images were collected every 5 minutes.

File Name: Supplementary Movie 3
Description: Fluorescence images show time-lapse series of a stable TUBGsgU2OS cell co-expressing a γ-tubulinresist and cell cycle chromobody (CCCRFP). The image series illustrates the accumulation of CCCRFP during S-phase execution. Images were collected every 5 minutes.

File Name: Supplementary Movie 4
Description: Fluorescence images show time-lapse series of a stable TUBGsgU2OS cell co-expressing a γ-tubulinA429-A432- A435 resist and cell cycle chromobody (CCCRFP). The image series illustrates the accumulation of CCCRFP and how mutations in the γ-tubulin PIP-motif affect the formation of CCCRFP foci during S-phase execution. Images were collected every 5 minutes.
