# Supplementary material for: The γ-tubulin meshwork assists in the recruitment of PCNA to chromatin in mammalian cells
Source: Commun Biol. 2021 Jun 22;4:767. doi: 10.1038/s42003-021-02280-1 (PMC8219688; doi:10.1038/s42003-021-02280-1)
Supplement: Supplementary file 9 — Reporting Summary [file 42003_2021_2280_MOESM9_ESM.pdf]

nature research

Corresponding author(s): Maria Alvarado Kristensson

Last updated by author(s): May 18, 2021

Reporting Summary

Nature Research wishes to improve the reproducibility of the work that we publish. This form provides structure for consistency and transparency in reporting. For further information on Nature Research policies, see our Editorial Policies and the Editorial Policy Checklist.

Statistics

For all statistical analyses, confirm that the following items are present in the figure legend, table legend, main text, or Methods section.

n/a	Confirmed	
☐	☒	The exact sample size (n) for each experimental group/condition, given as a discrete number and unit of measurement
☒	☐	A statement on whether measurements were taken from distinct samples or whether the same sample was measured repeatedly
☐	☒	The statistical test(s) used AND whether they are one- or two-sided<br>*Only common tests should be described solely by name; describe more complex techniques in the Methods section.*
☒	☐	A description of all covariates tested
☒	☐	A description of any assumptions or corrections, such as tests of normality and adjustment for multiple comparisons
☐	☒	A full description of the statistical parameters including central tendency (e.g. means) or other basic estimates (e.g. regression coefficient) AND variation (e.g. standard deviation) or associated estimates of uncertainty (e.g. confidence intervals)
☒	☐	For null hypothesis testing, the test statistic (e.g. F, t, r) with confidence intervals, effect sizes, degrees of freedom and P value noted<br>*Give P values as exact values whenever suitable.*
☒	☐	For Bayesian analysis, information on the choice of priors and Markov chain Monte Carlo settings
☒	☐	For hierarchical and complex designs, identification of the appropriate level for tests and full reporting of outcomes
☐	☒	Estimates of effect sizes (e.g. Cohen's d, Pearson's r), indicating how they were calculated

Our web collection on statistics for biologists contains articles on many of the points above.

Software and code

Policy information about availability of computer code

Data collection	Zeiss LSM 700 Axio Observer microscope, Ion proton system, 5500 W Series Genetic Analyzers
Data analysis	MEME-ChIP, PeakSeq, ImageJ, Zen,prisma, FlowJo, GEPIA software, Prism and excel.

For manuscripts utilizing custom algorithms or software that are central to the research but not yet described in published literature, software must be made available to editors and reviewers. We strongly encourage code deposition in a community repository (e.g. GitHub). See the Nature Research guidelines for submitting code & software for further information.

Data

Policy information about availability of data

All manuscripts must include a data availability statement. This statement should provide the following information, where applicable:

- Accession codes, unique identifiers, or web links for publicly available datasets
- A list of figures that have associated raw data
- A description of any restrictions on data availability

All accession codes are included in the article and in this form. All other data is available upon request.

Field-specific reporting

Life sciences study design

All studies must disclose on these points even when the disclosure is negative.

Sample size	The sample size was determined by the number of independent experiments that were performed.
Data exclusions	No data was excluded if the internal controls of the experiment were working.
Replication	Yes the experimental findings were reliably reproduced
Randomization	NA
Blinding	NA

Reporting for specific materials, systems and methods

We require information from authors about some types of materials, experimental systems and methods used in many studies. Here, indicate whether each material, system or method listed is relevant to your study. If you are not sure if a list item applies to your research, read the appropriate section before selecting a response.

Materials & experimental systems

n/a	Involved in the study	
☐	☒	Antibodies
☐	☒	Eukaryotic cell lines
☒	☐	Palaeontology and archaeology
☒	☐	Animals and other organisms
☒	☐	Human research participants
☐	☒	Clinical data
☒	☐	Dual use research of concern

Methods

n/a	Involved in the study	
☐	☒	ChIP-seq
☒	☐	Flow cytometry
☒	☐	MRI-based neuroimaging

Antibodies

Antibodies used	anti-GFP , Ran, Mcm5, Mcm6, RFC3, lamin B1, TCP1 beta, anti-gamma-tubulin, beta3-tubulin, Sox2, nucleolin, GCP2, polymerase delta, anti-pericentrin, anti-PCNA, anti-pSer139 H2AX and anti-α-tubulin
Validation	Antibodies against gamma-tubulin and PCNA were validated with sg-TUBG and sg-PCNA. Furthermore, for establishing the affinity of anti-gamma-tubulin antibody towards TUBG1 and TUBG2, the antibodies were tested in WB using cell lines that either expressed TUBG1 or TUBG2. In addition, shTUBG (which decreased the levels of gamma-tubulin by 50% was also used to assure the specificity of the antibodies used int the Chip-seq.

Eukaryotic cell lines

Policy information about cell lines

Cell line source(s)	ACC and H9-NSC (a gift from Dr. Karayan-Tapon).
Authentication	We assay their morphology, size and protein expression. In addition, the different cell lines require different growth media. The authentication of H9-NSC was carried out by eurofins genomics Europe Applied genomics, by using a PCR-single-locus-technology.
Mycoplasma contamination	Yes, all cell lines are routinely tested for micoplasma contamination.
Commonly misidentified lines (See ICLAC register)	The effects we study are not cell line specific. We study how PCNA is transported into the nuclear compartment in mammalian cells.

Clinical data

Policy information about clinical studies

All manuscripts should comply with the ICMJEguidelines for publication of clinical research and a completedCONSORT checklist must be included with all submissions.

Clinical trial registration	NA
Study protocol	NA
Data collection	Ref 445/07
Outcomes	NA

ChIP-seq

Data deposition

- [x] Confirm that both raw and final processed data have been deposited in a public database such as GEO.
- [x] Confirm that you have deposited or provided access to graph files (e.g. BED files) for the called peaks.

Data access links
May remain private before publication.

GSM2884568, GSM2884569, GSM2884570, GSM2884571, GSM2884576, GSM2884577, GSM2884578, GSM2884579, GSM2884584, GSM2884585, GSM2884586, GSM2884587, GSM2884588, GSM2884589, GSM2884590, GSM2884591, GSM2884572, GSM2884573, GSM2884574, GSM2884575, GSM2884580, GSM2884581, GSM2884582, GSM2884583, GSM4481346, GSM4481347, GSM4481348, GSM4481349, GSM4481350, GSM4481351, GSM4481352, GSM4481353, GSM4481354, GSM4481355, GSM4481356, GSM4481357, GSM4481358, GSM4481359, GSM4481360, GSM4481361, GSM4481362, GSM4481363, GSM4481364, GSM4481365, GSM4481366, GSM4481367,
GSM5277562, GSM5277563,GSM5277564,GSM5277565, GSM5277566, GSM5277567, GSM5277568, GSM5277569, GSM5277570, GSM5277571, GSM5277572, GSM5277573, GSM5277574, GSM5277575, GSM5277576, GSM5277577, GSM5277578, GSM5277579, GSM5277580, GSM5277581, GSM5277582, GSM5277583, GSM5277584, GSM5277585, GSM5277586, GSM5277587, GSM5277588, GSM5277589, GSM5277590, GSM5277591, GSM5277592, GSM5277593, GSM5277594, GSM5277595, GSM5277596, GSM5277597, GSM5277598, GSM5277599, GSM5277600, GSM5277601, GSM5277602, GSM5277603, GSM5277604, GSM5277605, GSM5277606, GSM5277607, GSM5277608, GSM5277609, GSM5277610, GSM5277611, GSM5277612, GSM5277613, GSM5277614, GSM5277615, GSM5277616, GSM5277617, GSM5277618, GSM5277619, GSM5277620, GSM5277621, GSM5277622, GSM5277623, GSM5277624, and GSM5277625

Files in database submission

MCF10Ashy_ytub_1.bedgraph, MCF10Ashy_ytub_2.bedgraph, MCF10Ashy_ytub_1.1.fastq.gz, MCF10Ashy_ytub_1.2.fastq.gz, MCF10Ashy_ytub_2.1.fastq.gz, MCF10Ashy_ytub_2.2.fastq.gz, MCF10A_WCE_1.1.fastq.gz, MCF10A_WCE_1.2.fastq.gz, MCF10A_WCE_2.1.fastq.gz, MCF10A_WCE_2.2.fastq.gz, MCF10Ashy_WCE_1.1.fastq.gz, MCF10Ashy_WCE_1.2.fastq.gz, MCF10Ashy_WCE_2.1.fastq.gz, MCF10Ashy_WCE_2.2.fastq.gz, MCF10A_ytub_1.1.fastq.gz, MCF10A_ytub_1.2.fastq.gz, MCF10A_ytub_2.1.fastq.gz, MCF10A_ytub_2.2.fastq.gz, MCF10A_ytub_1.bedgraph, MCF10A_ytub_2.bedgraph, MCF10A_PCNA_1.1.fastq.gz, MCF10A_PCNA_1.2.fastq.gz, MCF10A_PCNA_2.1.fastq.gz, MCF10A_PCNA_2.2.fastq.gz, MCF10A_PCNA_1.bedgraph, MCF10A_PCNA_2.bedgraph, MCF10Ashy_PCNA 1.1.fastq.gz, MCF10Ashy_PCNA 1.2.fastq.gz, MCF10Ashy_PCNA 2.1.fastq.gz, MCF10Ashy_PCNA 2.2.fastq.gz,
MCF10Ashy_PCNA_1.bedgraph,MCF10Ashy_PCNA_2.bedgraph, gamma_tubulin_without_shRNAi_1hour.narrowPeak, gamma_tubulin_without_shRNAi_2hour.narrowPeak, gamma_tubulin_with_shRNAi_1hour.narrowPeak
gamma_tubulin_with_shRNAi_2hour.narrowPeak, PCNA_without_shRNAi_1hour.narrowPeak, PCNA_without_shRNAi_2hour.narrowPeak, PCNA_with_shRNAi_1hour.narrowPeak,PCNA_with_shRNAi_2hour.narrowPeak
gTub_0h.bedgraph, gTub_3h.bedgraph, gTub_5h.bedgraph, gTub_7h.bedgraph, gTub_9h.bedgraph, gTub_G0_G1.bedgraph
MH2A_3h.bedgraph, MH2A_5h.bedgraph, PCNA_3h.bedgraph, PCNA_5h.bedgraph, p1.ugc_595_3_F3.csfasta, p1.ugc_595_4_F3.csfasta, p1.ugc_595_5_F3.csfasta, p1.ugc_599_1_F3.csfasta, p1.ugc_609_F3.csfasta, p1.ugc_599_7_F3.csfasta, p1.ugc_595_6_F3.csfasta, p1.ugc_595_7_F3.csfasta, p1.ugc_595_8_F3.csfasta, p1.ugc_595_9_F3.csfasta, p1.ugc_595_1_F3.csfasta, p1.ugc_595_2_F3.csfasta, p1.ugc_599_2_F3.csfasta, p1.ugc_613_1_F3.csfasta, p1.ugc_599_5_F3.csfasta, p1.ugc_599_8_F3.csfasta, p1.ugc_599_3_F3.csfasta, p1.ugc_613_2_F3.csfasta, p1.ugc_599_6_F3.csfasta, p1.ugc_599_9_F3.csfasta, p1.ugc_613_3_F3.csfasta, p1.ugc_613_4_F3.csfasta, p1.ugc_595_3_F3.qual, p1.ugc_595_4_F3.qual, p1.ugc_595_5_F3.qual, p1.ugc_599_1_F3.qual, p1.ugc_609_F3.qual, p1.ugc_599_7_F3.qual, p1.ugc_595_6_F3.qual, p1.ugc_595_7_F3.qual, p1.ugc_595_8_F3.qual, p1.ugc_595_9_F3.qual, p1.ugc_595_1_F3.qual, p1.ugc_595_2_F3.qual, p1.ugc_599_2_F3.qual, p1.ugc_613_1_F3.qual, p1.ugc_599_5_F3.qual, p1.ugc_599_8_F3.qual, p1.ugc_599_3_F3.qual, p1.ugc_613_2_F3.qual, p1.ugc_599_6_F3.qual, p1.ugc_599_9_F3.qual, p1.ugc_613_3_F3.qual, p1.ugc_613_4_F3.qual,

Genome browser session
(e.g. UCSC)

Gamma-tubulin Chip-seq data performed on MCF10A are available from the 2nd of April 2018. PCNA Chip-seq data performed on MCF10A and all Chip-seq data performed on U2OS cells will be available after acceptance of the manuscript.

Methodology

Replicates	Data performed on MCF10A, TUBG-sh-MCF10A, U2OS and TUBG-sh-U2OS cells are 2 replicates for each sample and input from each time point. Data performed on U2OS cells are two independent experiments for each sample. The different IPs were performed from the same lysate.
Sequencing depth	Data was single-end

1. ChIP-seq MCF10A ytub 1.1, total nr reads 35,614,032; length 170 bp
2. ChIP-seq MCF10A ytub 1.2, total nr reads 54,752,732; length 174 bp; mapped 1 and 2, 96%
3. ChIP-seq MCF10A ytub 2.1, total nr reads 59,349,805; length 170 bp
4. ChIP-seq MCF10A ytub 2.2, total nr reads 34,763,686; length 173 bp; mapped 3 and 4, 93%
5. ChIP-seq MCF10Ashy ytub 1.1, total nr reads 49,303,995; length 173 bp
6. ChIP-seq MCF10Ashy ytub 1.2, total nr reads 41,191,636; length 173 bp; mapped 5 and 6, 91%
7. ChIP-seq MCF10Ashy ytub 2.1, total nr reads 51,150,116; length 174 bp
8. ChIP-seq MCF10Ashy ytub 2.2, total nr reads 50,040,101; length 172 bp; mapped 7 and 8, 92%
9. ChIP-seq MCF10A WCE 1.1, total nr reads 38,280,988; length 168 bp
10. ChIP-seq MCF10A WCE 1.2, total nr reads 46,070,759; length 170 bp; mapped 17 and 18, 98%
11. ChIP-seq MCF10A WCE 2.1, total nr reads 34,624,632; length 172
12. ChIP-seq MCF10A WCE 2.2, total nr reads 46,811,661; length 173 bp; mapped 19 and 20, 98%
13. ChIP-seq MCF10Ashy WCE 1.1, total nr reads 45,371,407; length 172 bp
14. ChIP-seq MCF10Ashy WCE 1.2, total nr reads 35,820,820; length 171; mapped 21 and 22, 99%
15. ChIP-seq MCF10Ashy WCE 2.1, total nr reads 39,553,938; length 171 bp
16. ChIP-seq MCF10Ashy WCE 2.2, total nr reads ; mapped; length 170 bp; mapped 23 and 24, 98%
17. Chip-seq MCF10A PCNA 1.1, total nr reads 55,054,087 ; length 171 bp
18. Chip-seq MCF10A PCNA 1.2, total nr reads 37,795,180; length 173 bp; mapped 5 and 6, 99%

Antibodies

MCF10A, U2OS, and TUBG-sh-MCF10A cells: Anti-gamma-tubulin a mixture (1:1) of T3320 and T5192 from sigma; anti-macro histone 2A1 #4827 (Cell Signaling); anti-PCNA sc-7907 (Santa Cruz biotechnology). U2OS and TUBG-sh-U2OS cells: Anti-gamma-tubulin a mixture (1:1) of T3320 and T5192 from sigma; anti-Mcm5 ab17967 (Abcam); and anti-FoxM1 GTX102170 (Gene Tex) and anti-PCNA clone PC10 MAB424 (Sigma-Aldrich).

Peak calling parameters

MCF10A, and TUBG-sh-MCF10A cells
The software used to collect and initial analysis of the data was performed with Torrent Suite v5.0.2. In addition, we analyzed the Chip-seq data using Bowtie (version 1.0.0) (parameters: -m 1) for mapping and MACS for peak calling (version: 2.1.1) and PeakSeq (Version 1.31) using the default setting.
U2OS
we analyzed the Chip-seq data using BFAST (Version 0.7.0a) with the default setting for reads mapping and PeakSeq (Version 1.31) with the default setting for peak calling
U2OS and TUBG-sh-U2OS cells
Chromatin was sequenced using the NovaSeq S1 flowcell, PE50 bp and v1 sequencing chemistry and an Illumina NovaSeq 6000 instrument (NovaSeq control software v 1.7.0/ RTA v3.4.4). Peaks were identified using MACS2 (parameters: callpeak -B -g hs --nomodel --extsize 200 -q 0.05).

Data quality

MCF10A, and TUBG-sh-MCF10A cells
Low quality bases were trimmed using Trimmomatic (https://www.ncbi.nlm.nih.gov/pubmed/26911985, version 0.33, parameters: LEADING:3 TRAILING:3 SLIDINGWINDOW:4:15 MINLEN:25). This method is used to ensure data quality.
The number of peaks in FDR cutoff of 5% are:

Protein IP	Time point	shRNAi	# Peaks
gamma tubulin	1h	Without shRNAi	1,035
	1h	With shRNAi	296
	2h	Without shRNAi	1,547
	2h	With shRNAi	84
PCNA	1h	Without shRNAi	562
	1h	With shRNAi	290
	2h	Without shRNAi	2,768
	2h	With shRNAi	400

U2OS
The number of peaks in FDR cutoff of 1% are:

Protein IP	Time point	Peaks
Gamma tubulin	G0/G1	4449
	0h	9629
	3h	18090
	5h	30790
	7h	29544

	9h	1386
PCNA	3h	19375
	5h	28617
MH2A	3h	435537
	5h	46740

U2OS and TUBG-sh-U2OS cells
Quality control was performed on the raw sequencing data using FastQC (v0.11.9). Low quality bases were trimmed using Trimmomatic (version 0.35) (LEADING:3 TRAILING: 3 SLIDINGWINDOW:4:15 MINLEN:25). This method is used to ensure data quality.
The number of peaks in FDR cutoff of 5% was used to determining significant peaks.

Protein IP	Time point	shRNAi	# Peaks
TUBG	3h	Without shRNAi	104
TUBG	3h	With shRNAi	51
PCNA	3h	Without shRNAi	17
PCNA	3h	With shRNAi	25
MCM5	3h	Without shRNAi	132
FoxM1	3h	Without shRNAi	202
TUBG	7h	Without shRNAi	75
TUBG	7h	With shRNAi	42
PCNA	7h	Without shRNAi	17
PCNA	7h	With shRNAi	25
MCM5	7h	Without shRNAi	29
FoxM1	7h	Without shRNAi	123
TUBG	9h	Without shRNAi	153
TUBG	9h	With shRNAi	67
PCNA	9h	Without shRNAi	130
PCNA	9h	With shRNAi	155
MCM5	9h	Without shRNAi	109
FoxM1	9h	Without shRNAi	244
TUBG	Non-synchr.	Without shRNAi	102
TUBG	Non-synchr.	With shRNAi	94
PCNA	Non-synchr.	Without shRNAi	118
PCNA	Non-synchr.	With shRNAi	87
MCM5	Non-synchr.	Without shRNAi	98
FoxM1	Non-synchr.	Without shRNAi	129

Software

MCF10A, and TUBG-sh-MCF10A cells
The software used to collect and initial analysis of the data was performed with Torrent Suite v5.0.2. In addition, we analyzed the Chip-seq data using Bowtie (version 1.0.0) (parameters: -m 1) for mapping and MACS for peak calling (version: 2.1.1) using the default setting

U2OS
we analyzed the Chip-seq data using BFAST (Version 0.7.0a) with the default setting for reads mapping and PeakSeq (Version 1.31) with the default setting for peak calling

U2OS and TUBG-sh-U2OS cells
The software used to collect and initial analysis of the data was performed with NovaSeq control software v 1.7.0/ RTA v3.4.4. In addition, we analyzed the Chip-seq data using Bowtie 2 (version 2.1.0) (parameters: -k 1) for mapping and MACS2 for peak calling using the following setting: callpeak -B -g hs --nomodel --extsize 200 -q 0.05)
